# Publisher Correction: Copper adparticle enabled selective electrosynthesis of n-propanol

**DOI:** 10.1038/s41467-020-14883-z

**Published:** 2020-02-20

**Authors:** Jun Li, Fanglin Che, Yuanjie Pang, Chengqin Zou, Jane Y. Howe, Thomas Burdyny, Jonathan P. Edwards, Yuhang Wang, Fengwang Li, Ziyun Wang, Phil De Luna, Cao-Thang Dinh, Tao-Tao Zhuang, Makhsud I. Saidaminov, Shaobo Cheng, Tianpin Wu, Y. Zou Finfrock, Lu Ma, Shang-Hsien Hsieh, Yi-Sheng Liu, Gianluigi A. Botton, Way-Faung Pong, Xiwen Du, Jinghua Guo, Tsun-Kong Sham, Edward H. Sargent, David Sinton

**Affiliations:** 1 Department of Mechanical and Industrial Engineering, University of Toronto, 5 King’s College Road, Toronto, ON M5S 3G8 Canada; 2 Department of Electrical and Computer Engineering, University of Toronto, 10 King’s College Road, Toronto, ON M5S 3G4 Canada; 3 Institute of New-Energy Materials, School of Materials Science and Engineering, Tianjin University, 300072 Tianjin, China; 4 Hitachi High Technologies America, Inc., 22610 Gateway Center Drive, Suite 100, Clarksburg, MD 20871 USA; 5 Materials for Energy Conversion and Storage, Department of Chemical Engineering, Delft University of Technology, 2629 HZ Delft, The Netherlands; 6 Department of Materials Science and Engineering, University of Toronto, 194 College Street, Toronto, ON M5S 3E4 Canada; 7 Canadian Center for Electron Microscopy, McMaster University, Hamilton, ON L8S 4M1 Canada; 8 Advanced Photon Source, Argonne National Laboratory, Lemont, IL 60439 USA; 9 Science Division, Canadian Light Source Inc., 44 Innovation Boulevard, Saskatoon, SK S7N 2V3 Canada; 10 Advanced Light Source, Lawrence Berkeley National Laboratory, Berkeley, CA 94720 USA; 11 Department of Physics, Tamkang University, 151 Yingzhuan Road, Tamsui District, 25137 New Taipei City ROC, Taiwan; 12 Department of Chemistry, University of Western Ontario, 1151 Richmond Street, London, ON N6A 5B7 Canada

**Keywords:** Electrocatalysis, Energy, Electrocatalysis

Correction to: *Nature Communications* 10.1038/s41467-018-07032-0, published online 5 November 2018.

The original version of this Article incorrectly omitted the received/accepted dates of Received: 05 June 2018; Accepted: 10 October 2018. This has now been corrected in the PDF and HTML versions of the Article.

