# Supplementary material for: Copper adparticle enabled selective electrosynthesis of n-propanol
Source: Nat Commun. 2018 Nov 5;9:4614. doi: 10.1038/s41467-018-07032-0 (PMC6218481; doi:10.1038/s41467-018-07032-0)
Supplement: Supplementary file 1 — Supplementary Information [file 41467_2018_7032_MOESM1_ESM.pdf]

Copper adparticle enabled selective electrosynthesis of n-propanol

Jun Li[1,2†], Fanglin Che[2†], Yuanjie Pang[1,2†], Chengqin Zou[2,3†], Jane Y. Howe[4], Thomas Burdyny[1,5], Jonathan P. Edwards[1], Yuhang Wang[2], Fengwang Li[2], Ziyun Wang[2], Phil De Luna[6], Cao-Thang Dinh[2], Tao-Tao Zhuang[2], Makhsud I. Saidaminov[2], Shaobo Cheng[7], Tianpin Wu[8], Y. Zou Finfrock[8,9], Lu Ma[8], Shang-Hsien Hsieh[10,11], Yi-Sheng Liu[10], Gianluigi A. Botton[7], Way-Faung Pong[11], Xiwen Du[3], Jinghua Guo[10], Tsun-Kong Sham[12], Edward H. Sargent[2]* & David Sinton[1]*

[1]*Department of Mechanical and Industrial Engineering, University of Toronto, 5 King's College Road, Toronto, Ontario, M5S 3G8, Canada.*
[2]*Department of Electrical and Computer Engineering, University of Toronto, 10 King's College Road, Toronto, Ontario, M5S 3G4, Canada.*
[3]*Institute of New-Energy Materials, School of Materials Science and Engineering, Tianjin University, Tianjin, 300072, China.*
[4]*Hitachi High Technologies America, Inc., 22610 Gateway Center Drive, Suite 100, Clarksburg, Maryland 20871, USA*
[5]*Materials for Energy Conversion and Storage, Department of Chemical Engineering, Delft University of Technology, 2629, HZ Delft, The Netherlands*
[6]*Department of Materials Science and Engineering, University of Toronto, 194 College Street, Toronto, Ontario, M5S 3E4, Canada*
[7]*Canadian Center for Electron Microscopy, McMaster University, Hamilton, Ontario, L8S 4M1, Canada*
[8]*Advanced Photon Source, Argonne National Laboratory, Lemont, Illinois, 60439, United States*
[9]*Science Division, Canadian Light Source Inc., 44 Innovation Boulevard, Saskatoon, Saskatchewan S7N 2V3, Canada*
[10]*Advanced Light Source, Lawrence Berkeley National Laboratory, Berkeley, California, 94720, United States*
[11]*Department of Physics, Tamkang University, 151 Yingzhuan Road, Tamsui District, New Taipei City, 25137, Taiwan, The Republic of China*
[12]*Department of Chemistry, University of Western Ontario, 1151 Richmond Street, London, Ontario, N6A 5B7, Canada*
[†] These authors contributed equally to this work.
[(*)] Correspondence and requests for materials should be addressed to Edward H. Sargent (ted.sargent@utoronto.ca) (E.H.S) and David Sinton (sinton@mie.utoronto.ca) (D. S.)

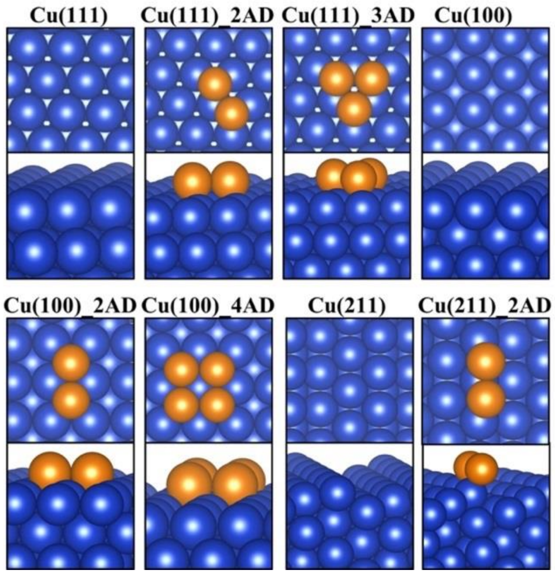

Supplementary Fig. 1. The optimized geometries of various Cu surfaces for examining the CO=CO dimerization and C_1-C_2 coupling during CO-RR.

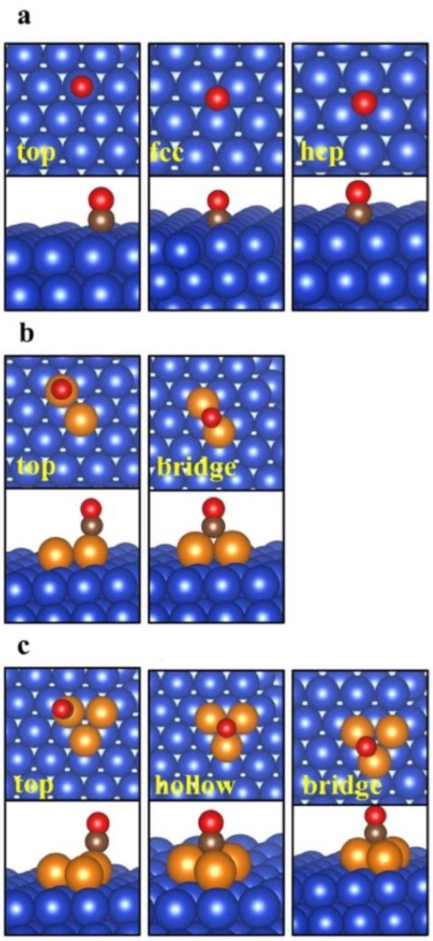

Supplementary Fig. 2. CO adsorptions on Cu(111) in the presence and absence of adparticles. a-c, The optimized geometries of CO adsorption on Cu(111) (**a**), Cu(111)_2AD (**b**) and Cu(111)_3AD (**c**).

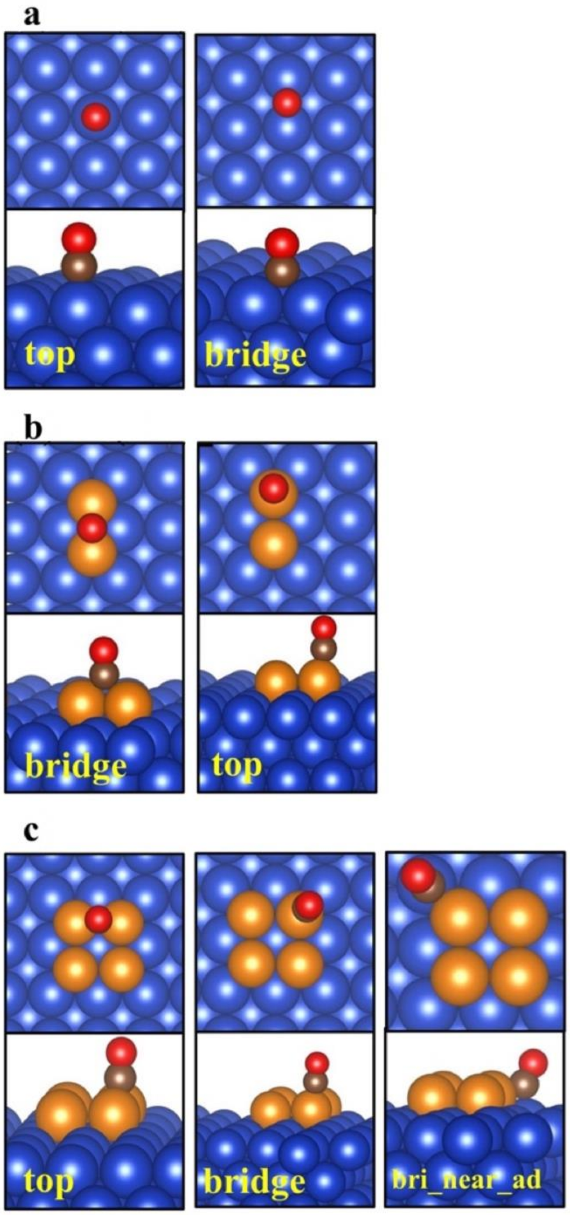

Supplementary Fig. 3. CO adsorptions on Cu(100) in the presence and absence of adparticles. a-c, The optimized geometries of CO adsorption on Cu(100) (**a**), Cu(100)_2AD (**b**) and Cu(100)_4AD (**c**).

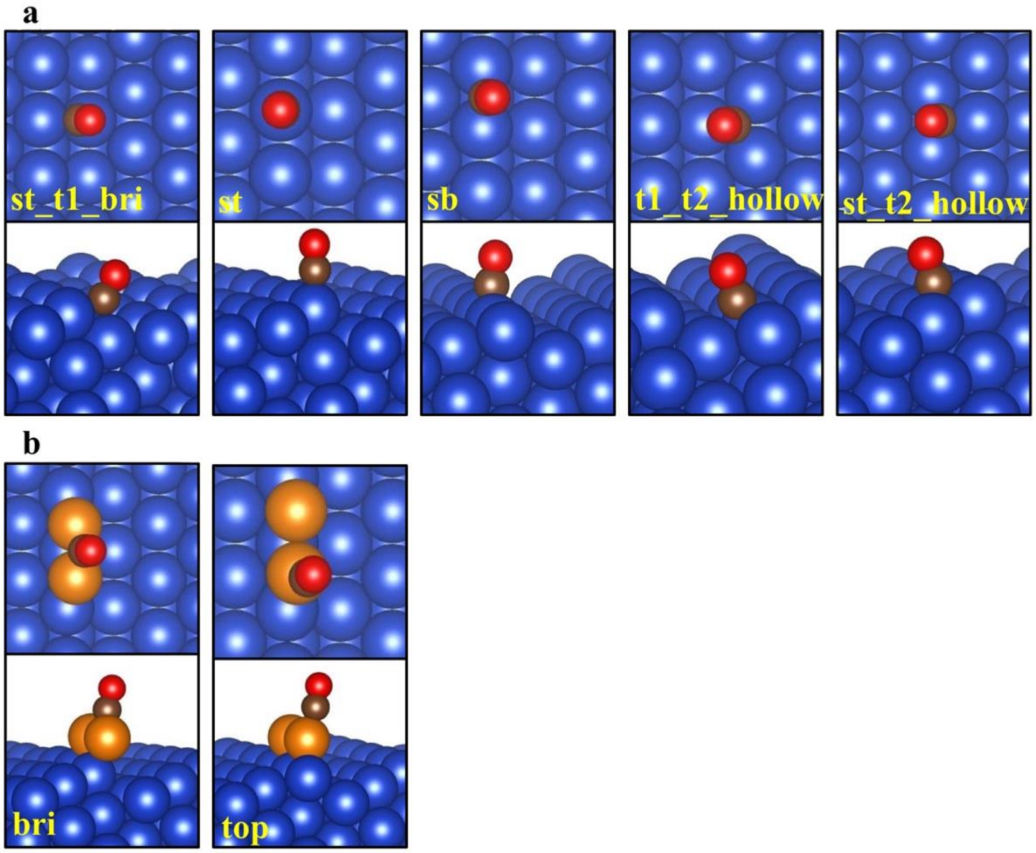

Supplementary Fig. 4. CO adsorptions on Cu(211) in the presence and absence of adparticles. a, b, The optimized geometries of CO adsorption on Cu(211) (**a**) and Cu(211)_2AD (**b**).

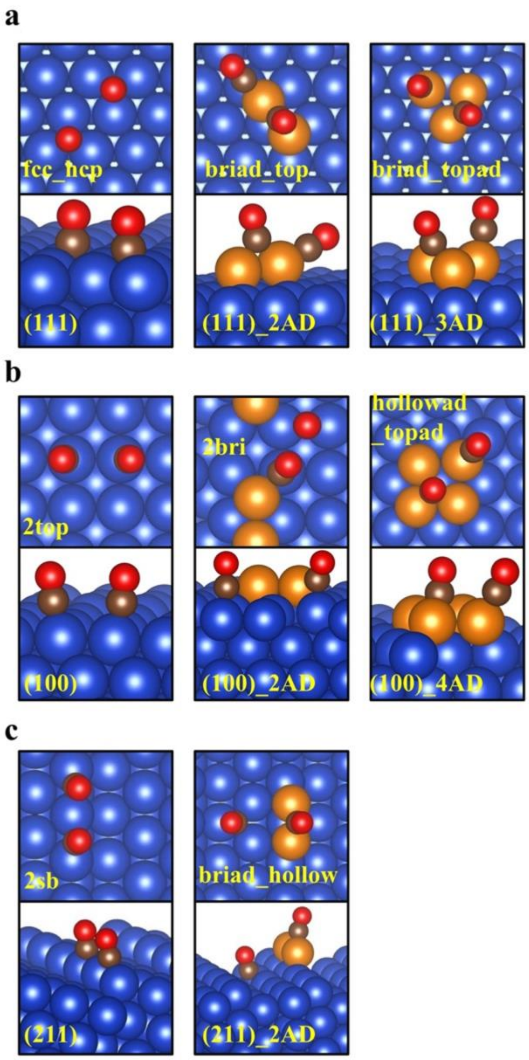

Supplementary Fig. 5. 2CO co-adsorptions on various Cu surfaces. a-c, The optimized geometries of 2CO co-adsorption on Cu(111) (**a**), Cu(100) (**b**) and Cu(211) (**c**) surfaces in the presence and absence of Cu adparticles.

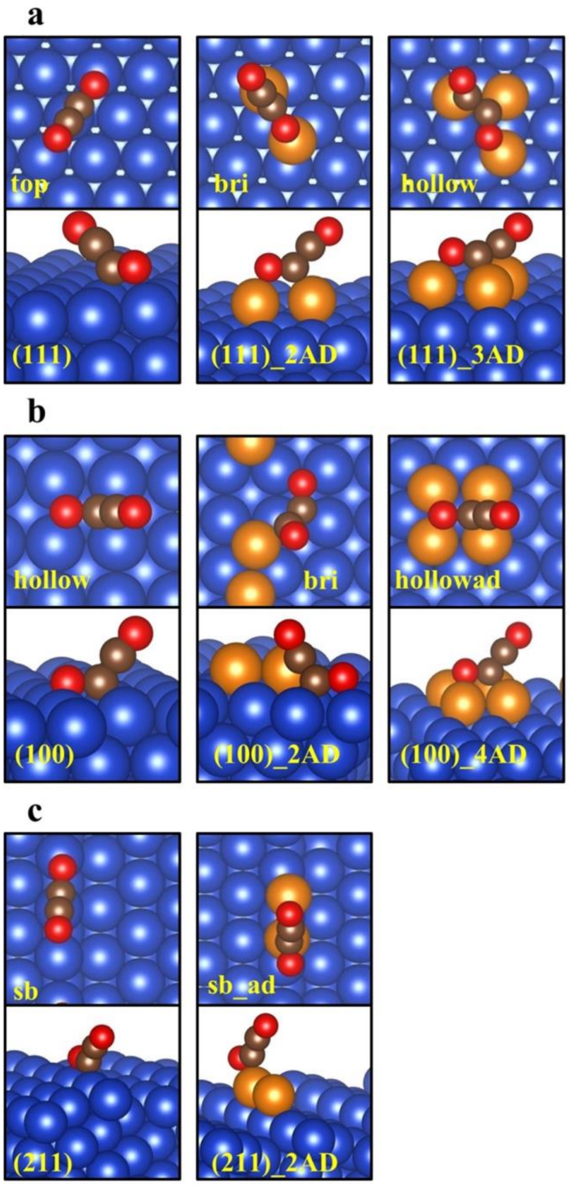

Supplementary Fig. 6. *OCCO adsorptions on various Cu surfaces. a-c, The optimized geometries of *OCCO adsorption on Cu(111) (**a**), Cu(100) (**b**) and Cu(211) (**c**) surfaces in the presence and absence of Cu adparticles.

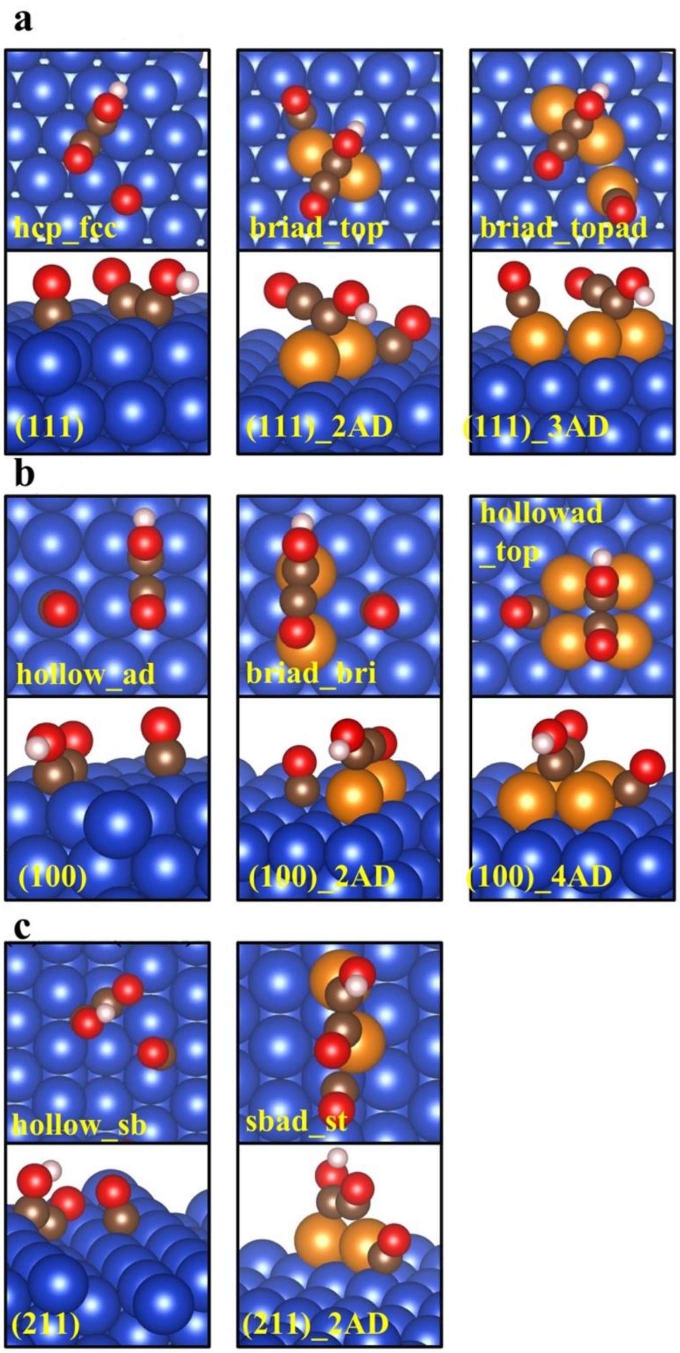

Supplementary Fig. 7. *CO and *OCCOH co-adsorptions on various Cu surfaces. a-c, The optimized geometries of *CO and *OCCOH co-adsorption on Cu(111) (**a**), Cu(100) (**b**) and Cu(211) (**c**) surfaces in the presence and absence of Cu adparticles.

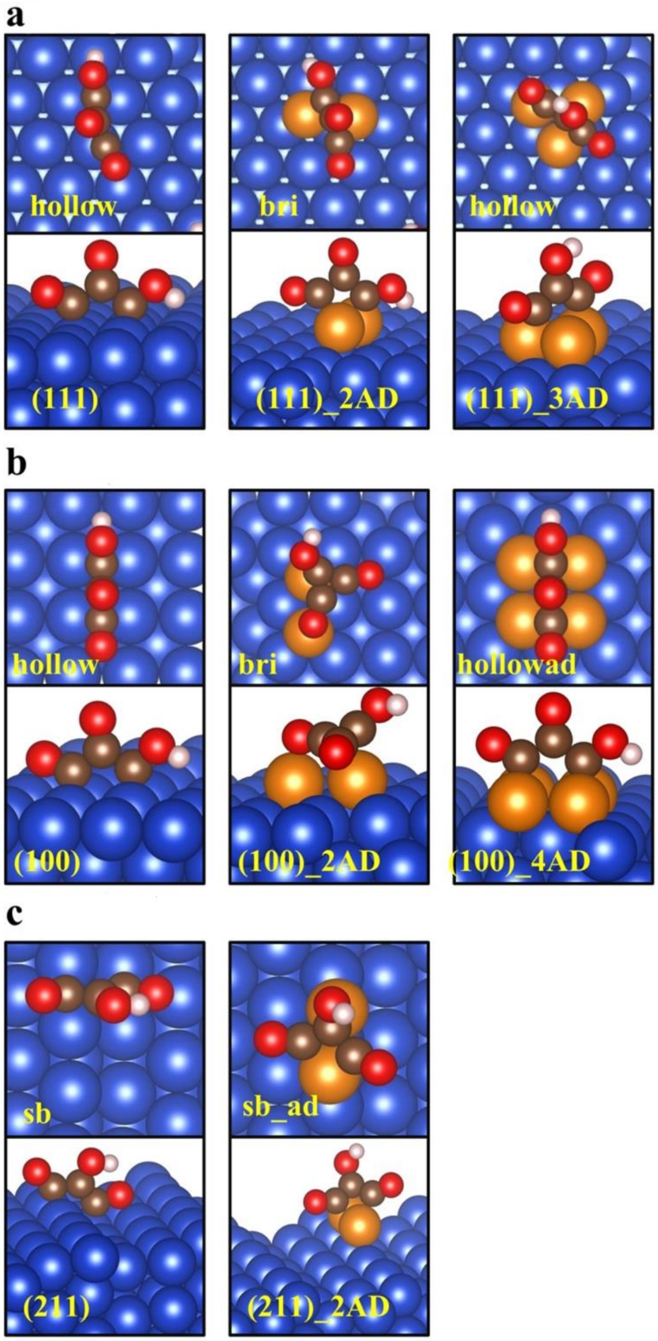

Supplementary Fig. 8. *OCOCCOH adsorptions on various Cu surfaces. a-c, The optimized geometries of *OCOCCOH adsorption on Cu(111) (**a**), Cu(100) (**b**) and Cu(211) (**c**) surfaces in the presence and absence of Cu adparticles.

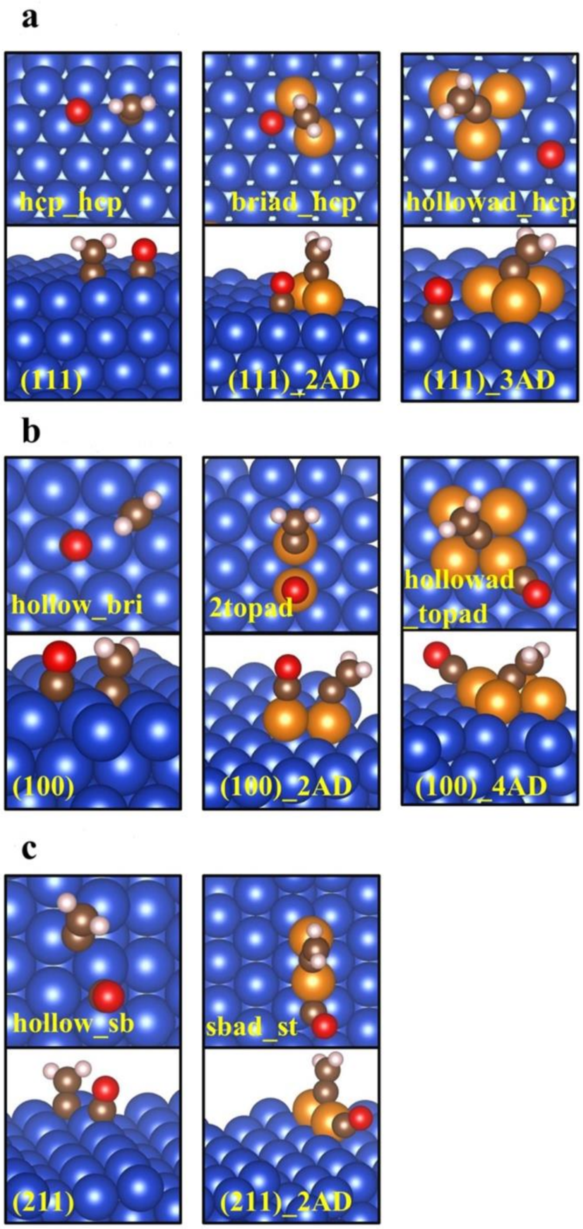

Supplementary Fig. 9. *CO and *CCH_2 co-adsorptions on various Cu surfaces. a-c, The optimized geometries of *CO and *CCH_2 co-adsorption on Cu(111) (**a**), Cu(100) (**b**) and Cu(211) (**c**) surfaces in the presence and absence of Cu adparticles.

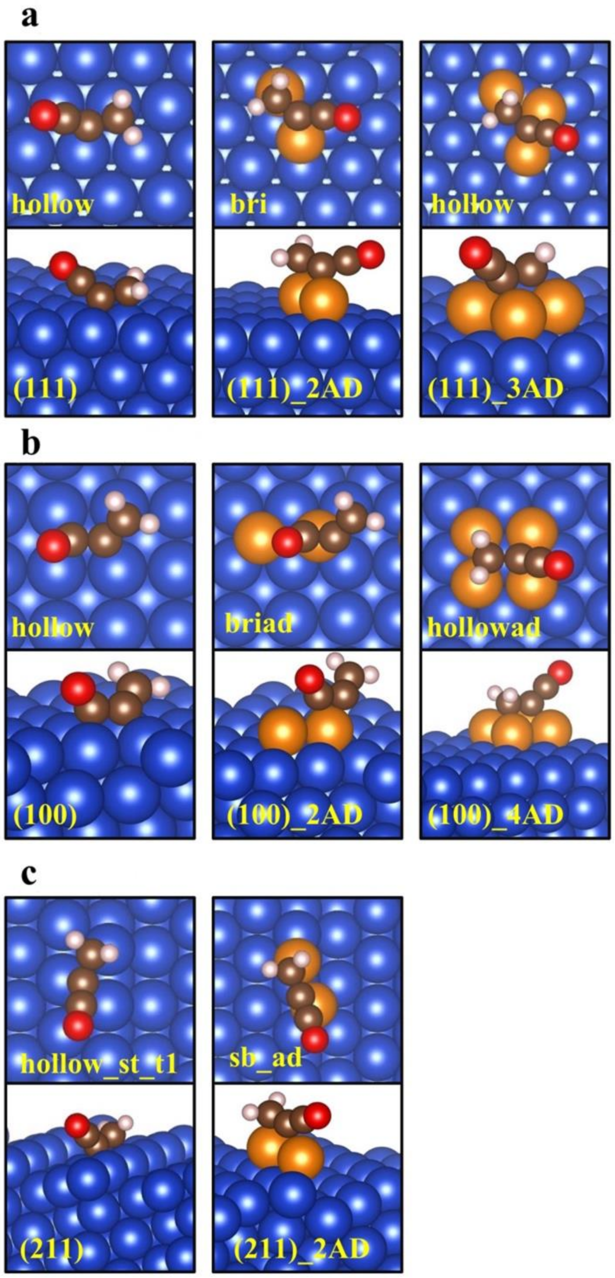

Supplementary Fig. 10. *$OCCCH_2$ adsorptions on various Cu surfaces. a-c, The optimized geometries of *$OCCCH_2$ adsorption on Cu(111) (**a**), Cu(100) (**b**) and Cu(211) (**c**) surfaces in the presence and absence of Cu adparticles.

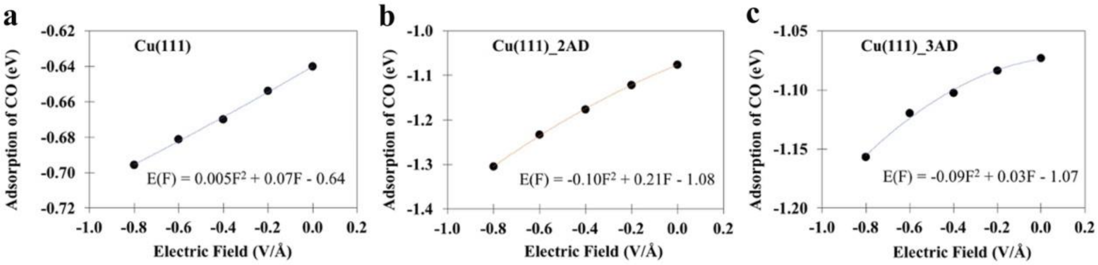

Supplementary Fig. 11. The applied field effects on the adsorption energies of CO over various Cu(111) sites. a, Cu(111) at a fcc site. **b**, Cu(111) with two Cu adatoms at a top site. **c,** Cu(111) with three Cu adatoms at a top site. The adsorption energies of the CO molecule on the Cu surface at their most favourable adsorption sites monotonically increased as we increased the applied field from 0 to 0.8 V/Å.

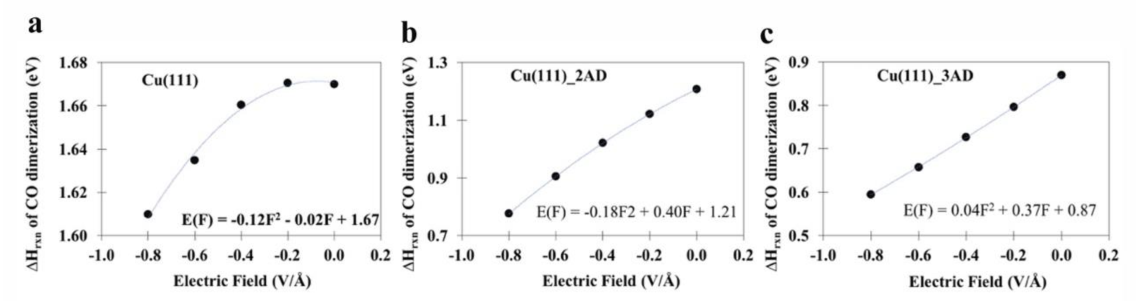

Supplementary Fig. 12. The applied field effects on the reaction energy of CO dimerization over various Cu(111) sites. a, Cu(111). **b**, Cu(111) with two Cu adatoms. **c,** Cu(111) with three Cu adatoms. The reaction energies of CO dimerization on the Cu surface monotonically decreased as we increased the applied field from 0 to 0.8 V/Å.

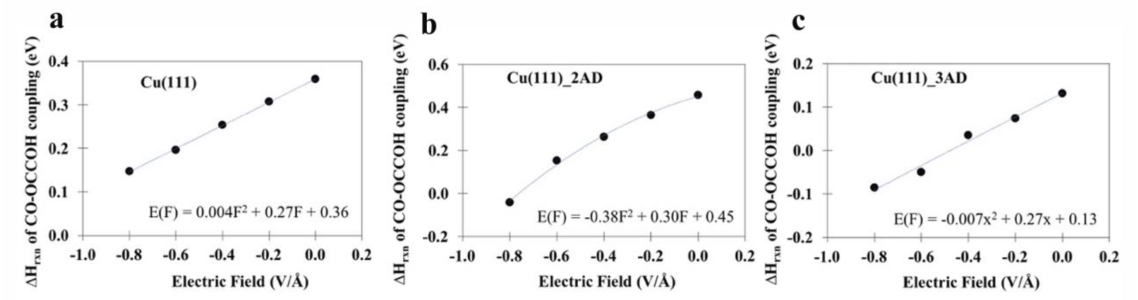

Supplementary Fig. 13. The applied field effects on the reaction energy of CO-OCCOH coupling over various Cu(111) sites. a, Cu (111). **b,** Cu(111) with two Cu adatoms. **c,** Cu(111) with three Cu adatoms. The reaction energies of CO-OCCOH on the Cu surface monotonically decreased as we increased the applied field from 0 to 0.8 V/Å.

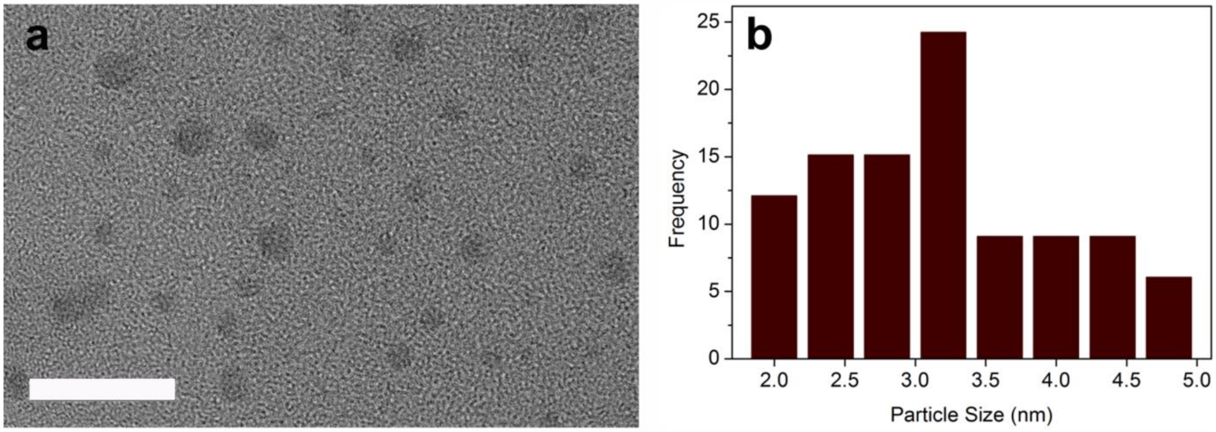

Supplementary Fig. 14. Characterizations of Cu adparticles. a, TEM image of Cu adparticles detached from the Cu backbone. The scale bar is 20 nm. **b,** Adparticle size distribution, a mean size of ~3.2 nm is determined.

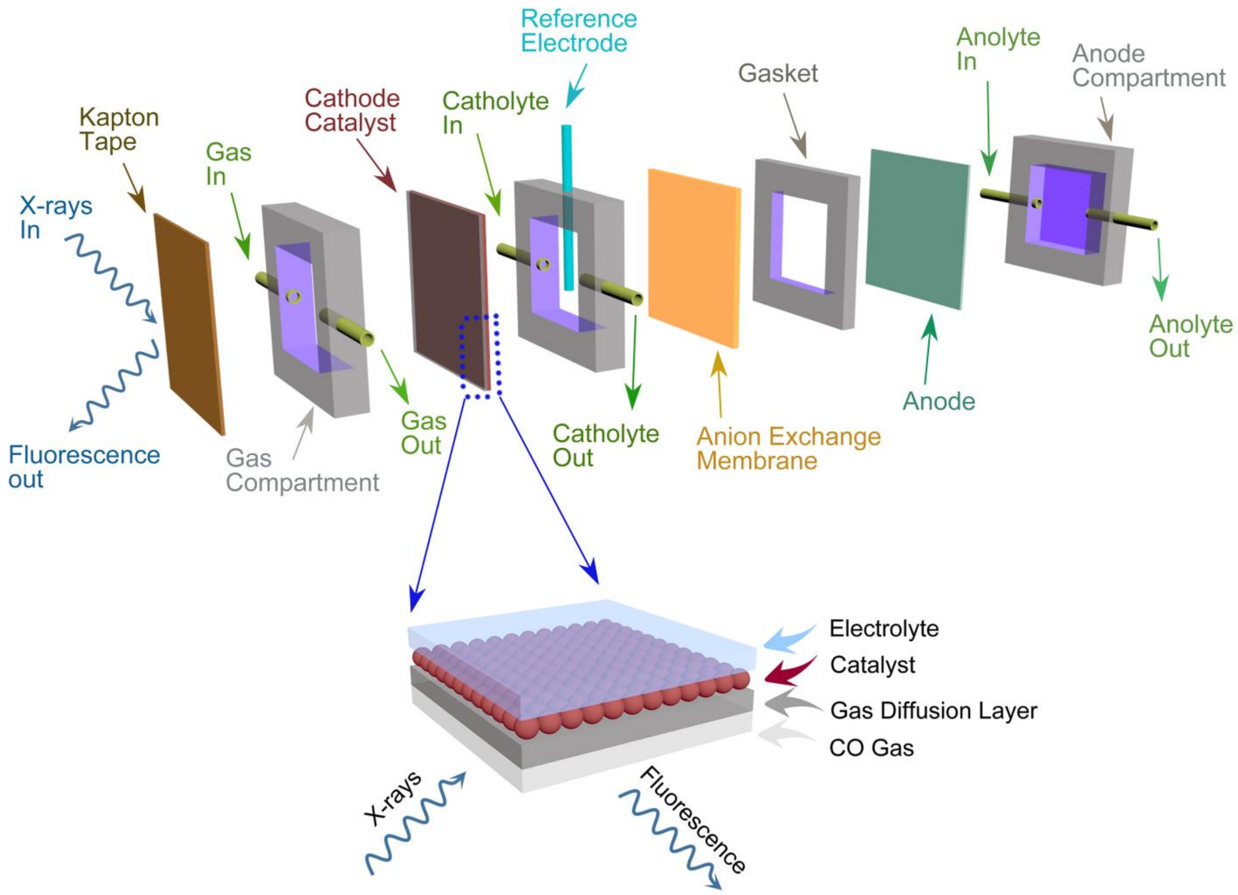

Supplementary Fig. 15. Explosive schematic view of flow cell and enlarged view of GDE for operando hXAS measurement.

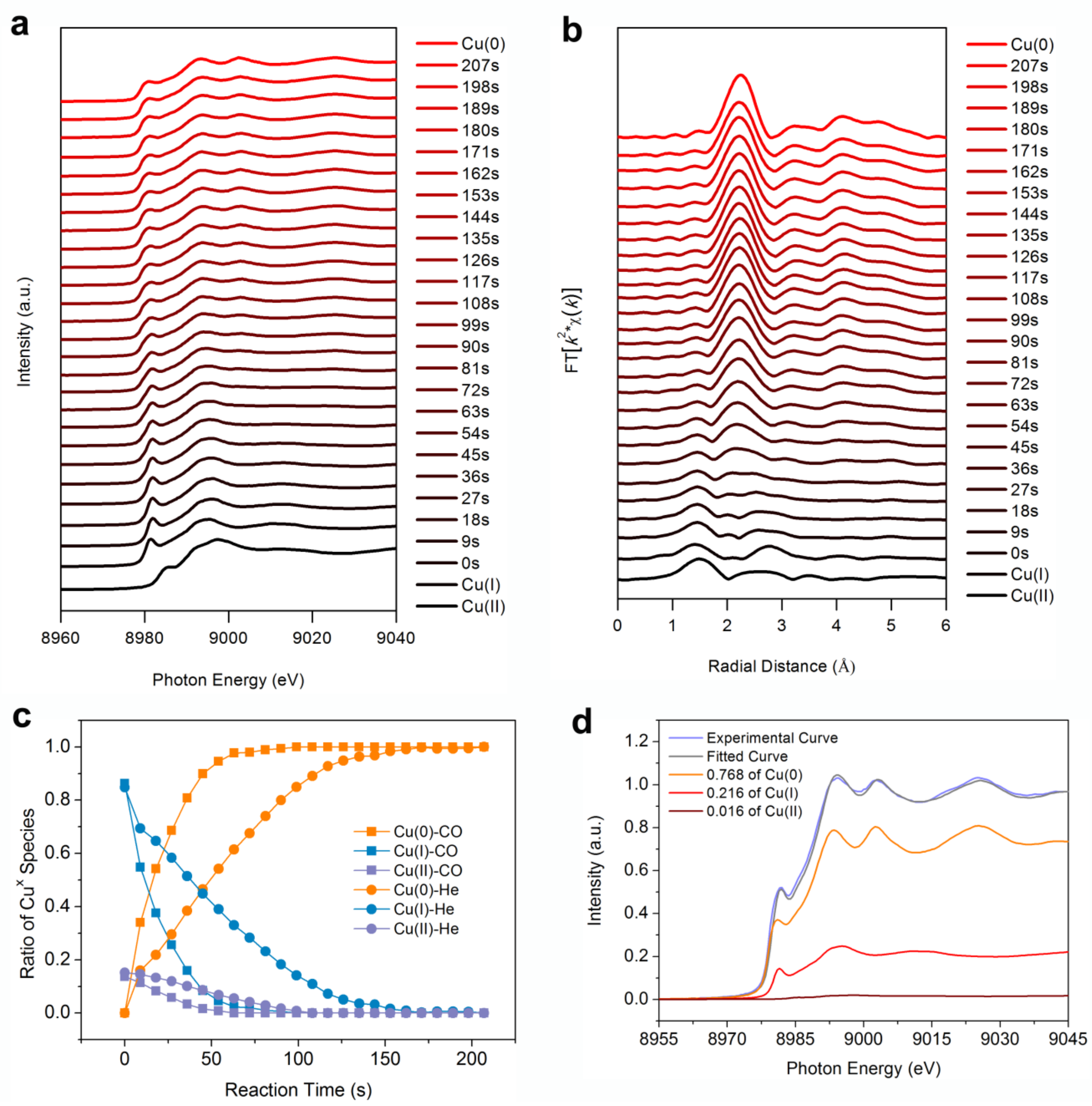

Supplementary Fig. 16. Operando hXAS analysis. a, b, Operando Cu K-edge hXAS analysis of oxide pre-catalysts derivation under a He (inert) gas condition. **c,** Calculated ratio of Cu oxidation states of derived Cu over the course of oxide reduction at -0.44 V vs. RHE in 1M KOH, under either a CO or He gas condition determined by linear combination fittings of their corresponding Cu K-edge hXAS. **d,** A quantitative linear combination fitting example of the Cu K-edge hXAS.

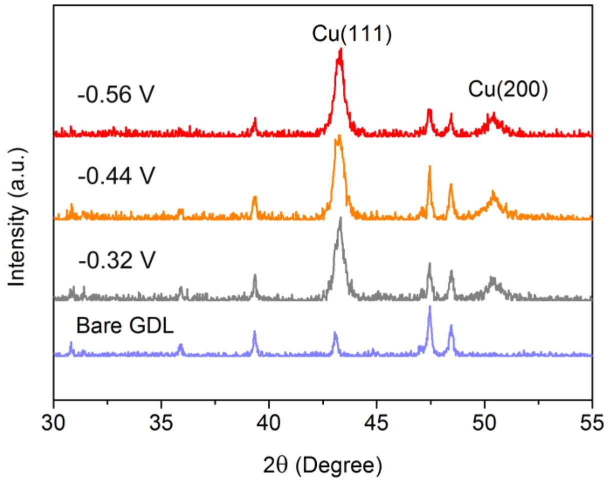

Supplementary Fig. 17. Ex-situ XRD spectra of bare gas diffusion layer (GDL) and Cu adparticle catalysts under CO gas at different applied potentials.

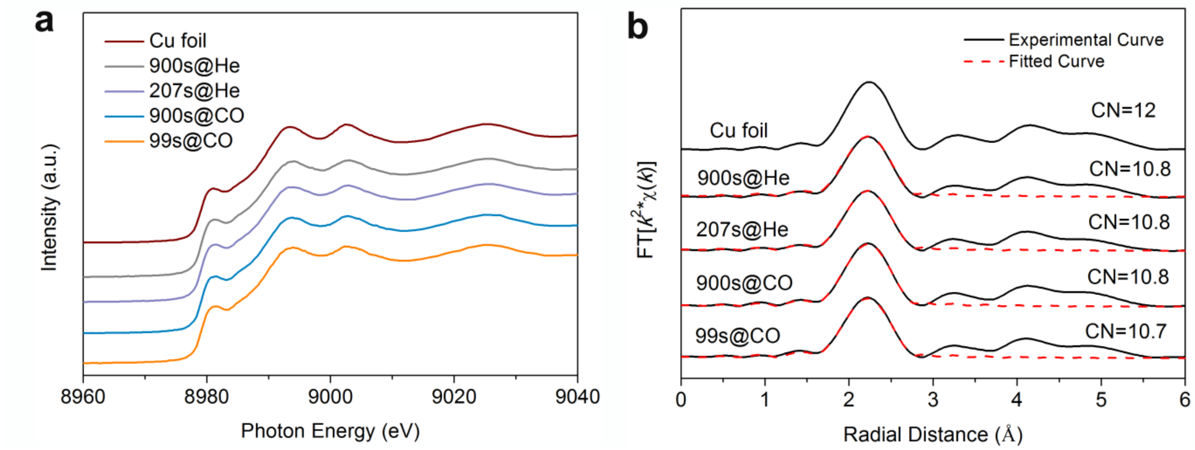

Supplementary Fig. 18. Operando hXAS analysis. a, Operando Cu K-edge hXAS of derived Cu species under CO (collected at 99s and 900s) and He (collected at 207s and 900s) gas conditions in comparison with that of Cu foil measured ex-situ. **b,** Operando Cu K-edge EXAFS plotted in R space and associated simulations at the first shell. Note that each spectrum was collected with a duration of 9s. The first shell fitting was conducted. $S_0/\Delta\sigma^2$ values of 0.9/0.00865 for Cu were determined from the Cu K-edge hXAS of Cu foil (CN = 12), which were directly applied to the Cu K-edge hXAS fittings of various Cu species.

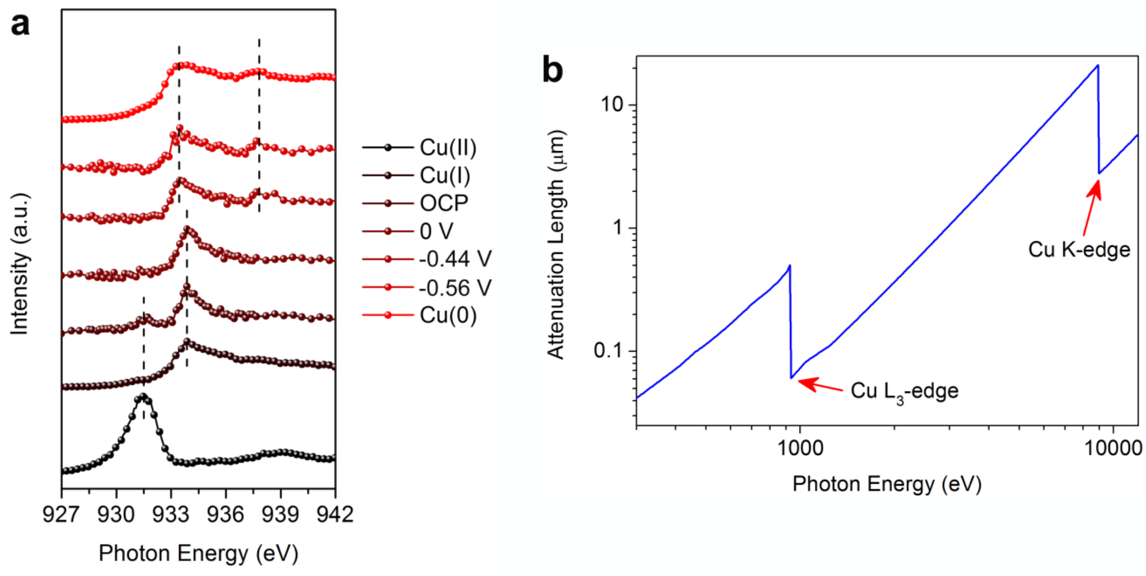

Supplementary Fig. 19. In-situ sXAS analysis. a, In-situ Cu L_3-edge sXAS of the Cu adparticle catalysts at various applied potentials (OCP represents the open circuit potential) as well as the Cu L_3-edge sXAS of copper(II) oxide, copper(I) oxide and metallic Cu standards measured ex-situ for comparison. **b,** Calculated X-ray attenuation length vs. photon energy in metallic Cu (density = 8.96 g cm^{-3}). The incident angle is 45 degree (http://henke.lbl.gov/optical_constants/atten2.html).

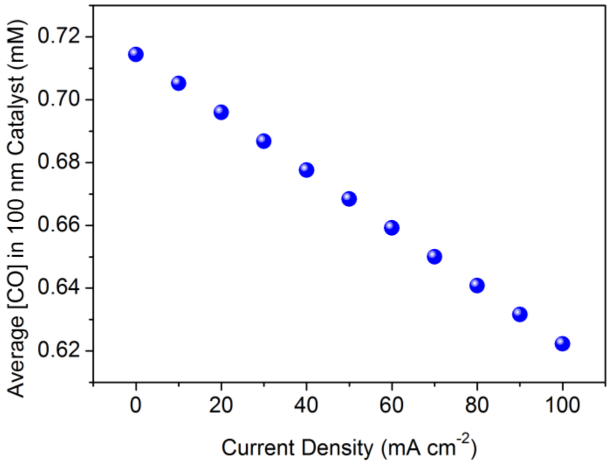

Supplementary Fig. 20. Modeling of the average [CO] in 100 nm catalyst at 1 atm under CO gas over the change of applied current densities (i.e., the change of applied potentials) in 1 M KOH.

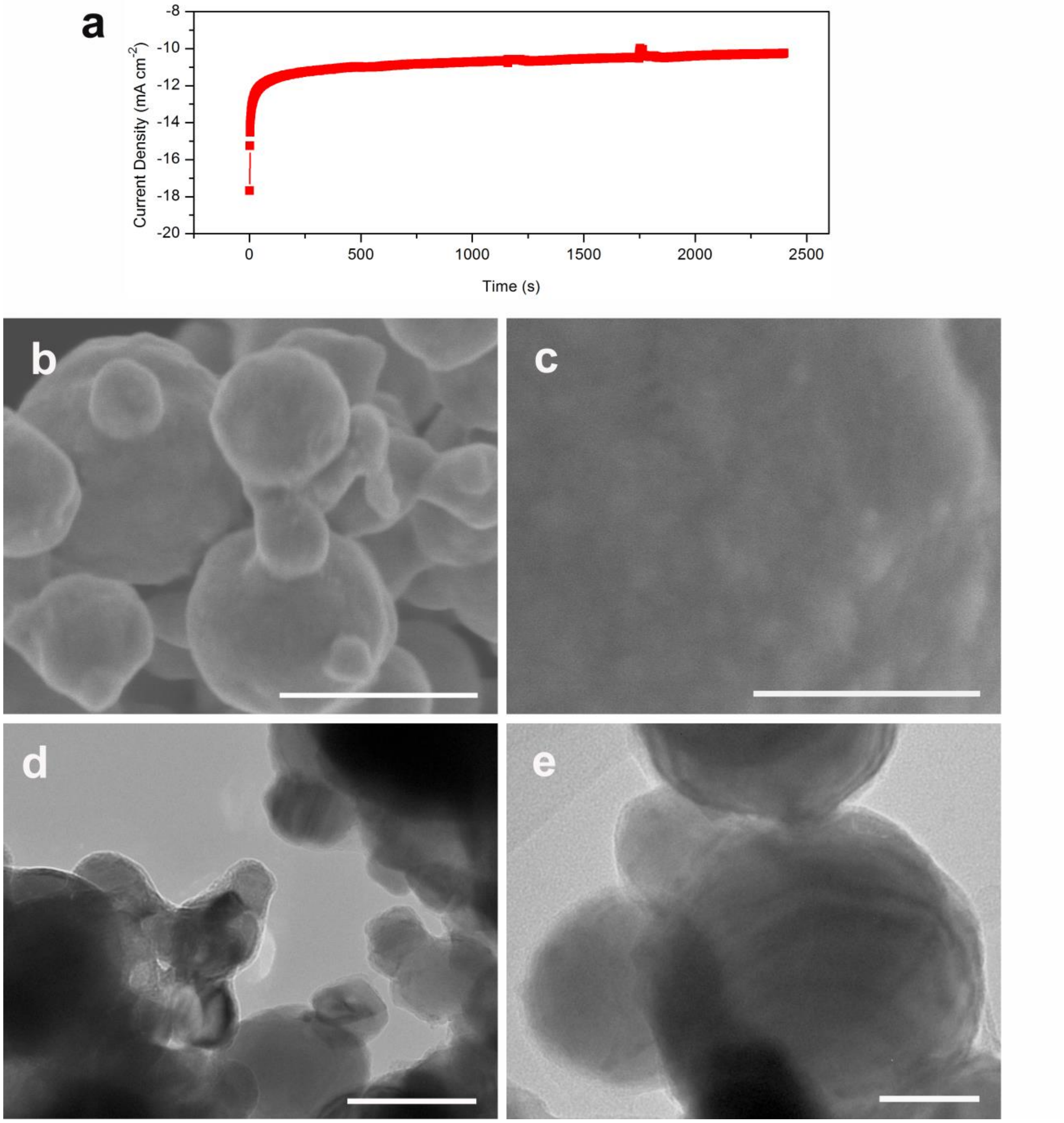

Supplementary Fig. 21. Characterizations of Cu nanoparticle control. a, *j-t* curve of Cu NP electrocatalysts at -0.44V in 1M KOH. **b, c,** SEM images of Cu NP electrocatalysts after CO-RR. The scale bars are 200 nm in (**b**) and 50 nm in (**c**). **d, e,** TEM examination of Cu NP electrocatalysts after CO-RR. The scale bars are 100 nm in (**d**) and 50 nm in (**e**).

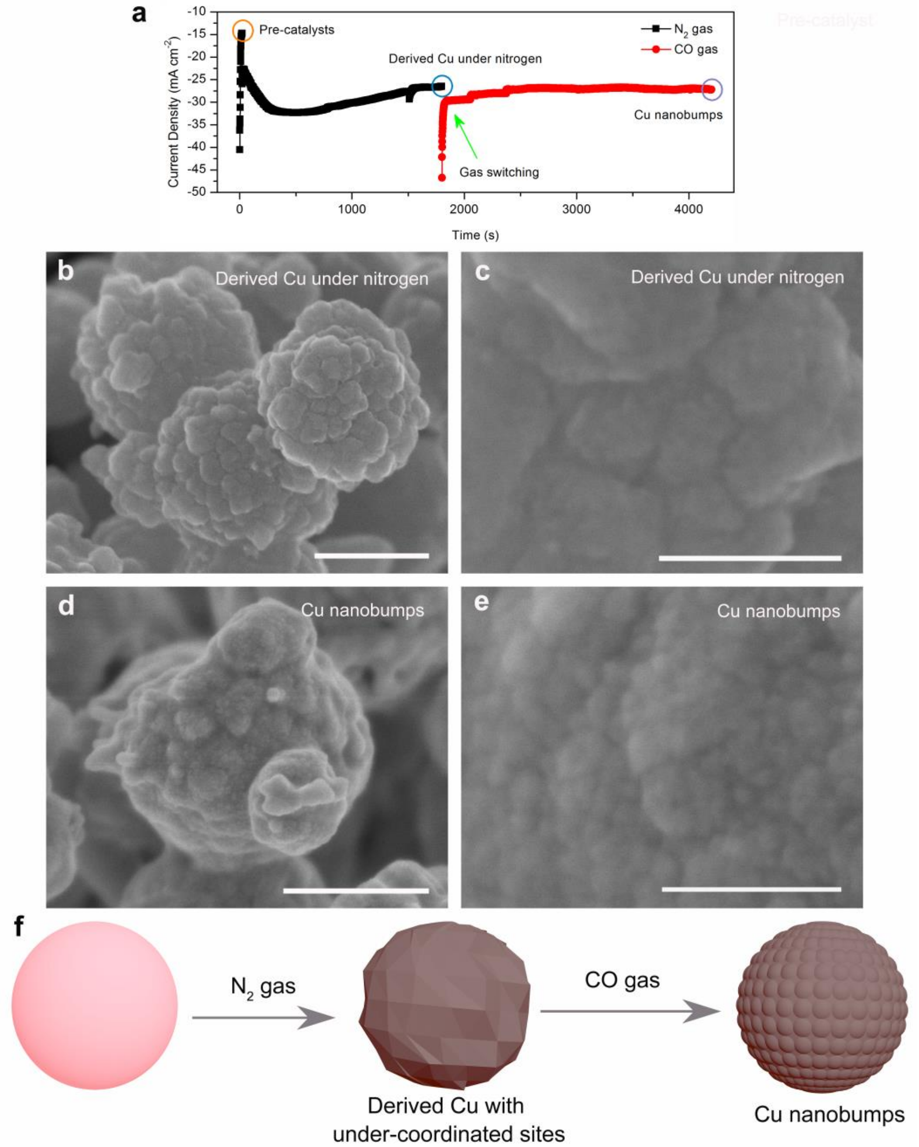

Supplementary Fig. 22. Characterizations of Cu nanobump control. a, *j*-*t* curve of Cu NB electrocatalysts at -0.44V in 1M KOH. **b, c,** SEM images of derived Cu under a N_2 gas condition at -0.44V in 1M KOH. **d, e,** SEM images of Cu NB electrocatalysts after a long duration of CO-RR at -0.44V in 1M KOH. The scale bars are 200 nm in (**b**) and (**d**), 50 nm in (**c**) and (**e**). **f,** Schematic illustration of the morphology evolution of derived-Cu catalysts at different reaction conditions.

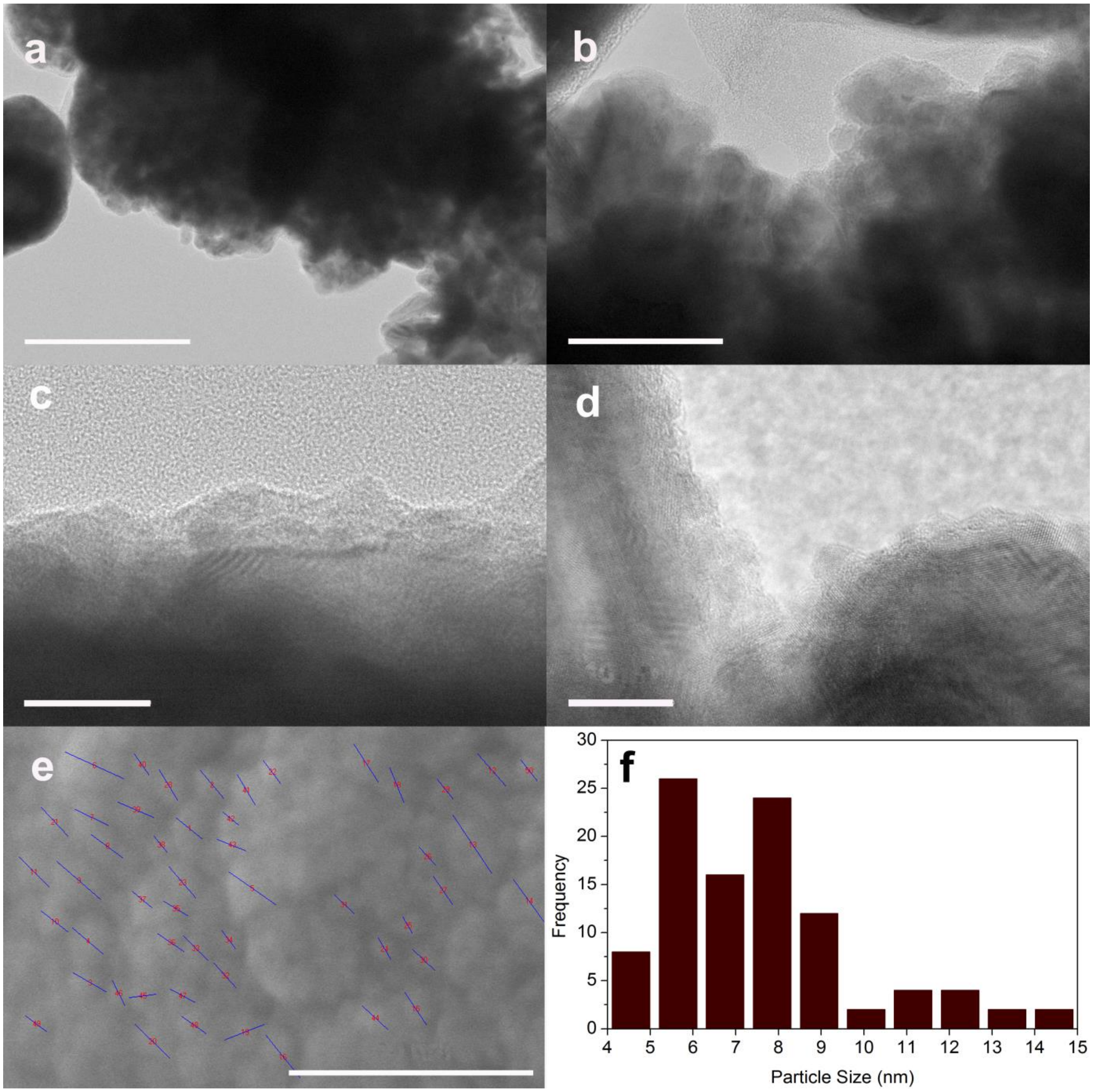

Supplementary Fig. 23. Characterizations of Cu nanobump control. a-d, TEM images of Cu NB electrocatalysts after a long duration of CO-RR reaction at -0.44V in 1M KOH. The scale bars are 200 nm in (**a**), 50 nm in (**b**), 20 nm in (**c**) and 10 nm in (**d**). **e, f,** Estimation of the nanobump size distribution. The scale bar is 50 nm in (**e**).

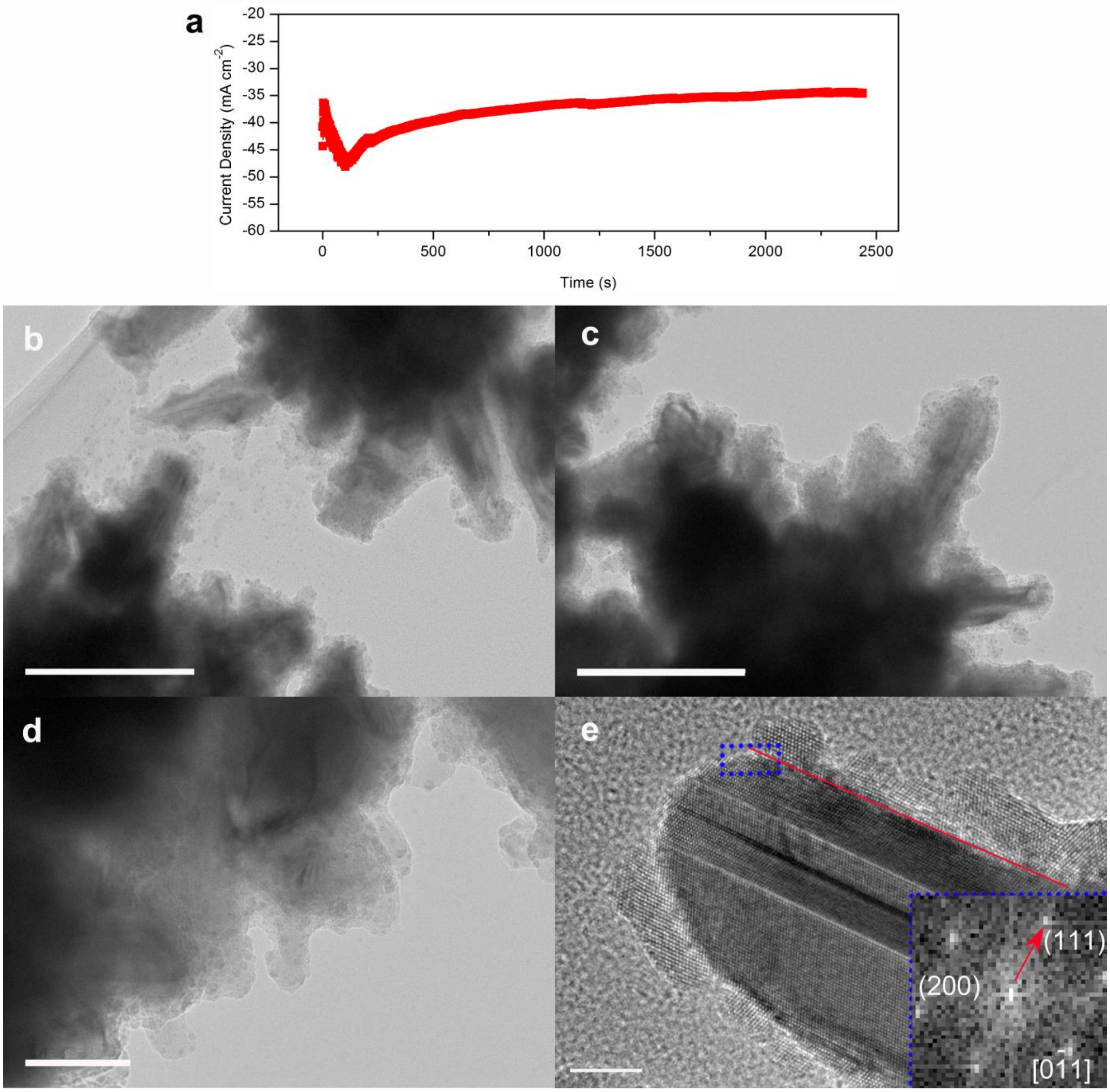

Supplementary Fig. 24. Characterizations of Cu adparticle. a, *j-t* curve of Cu AD electrocatalysts at -0.44V in 1M KOH. **b-e,** Morphology examination of Cu AD electrocatalysts after a long duration of CO-RR. The scale bars are 200 nm in (**b**) and (**c**), 50 nm in (**d**) and 5 nm in (**e**).

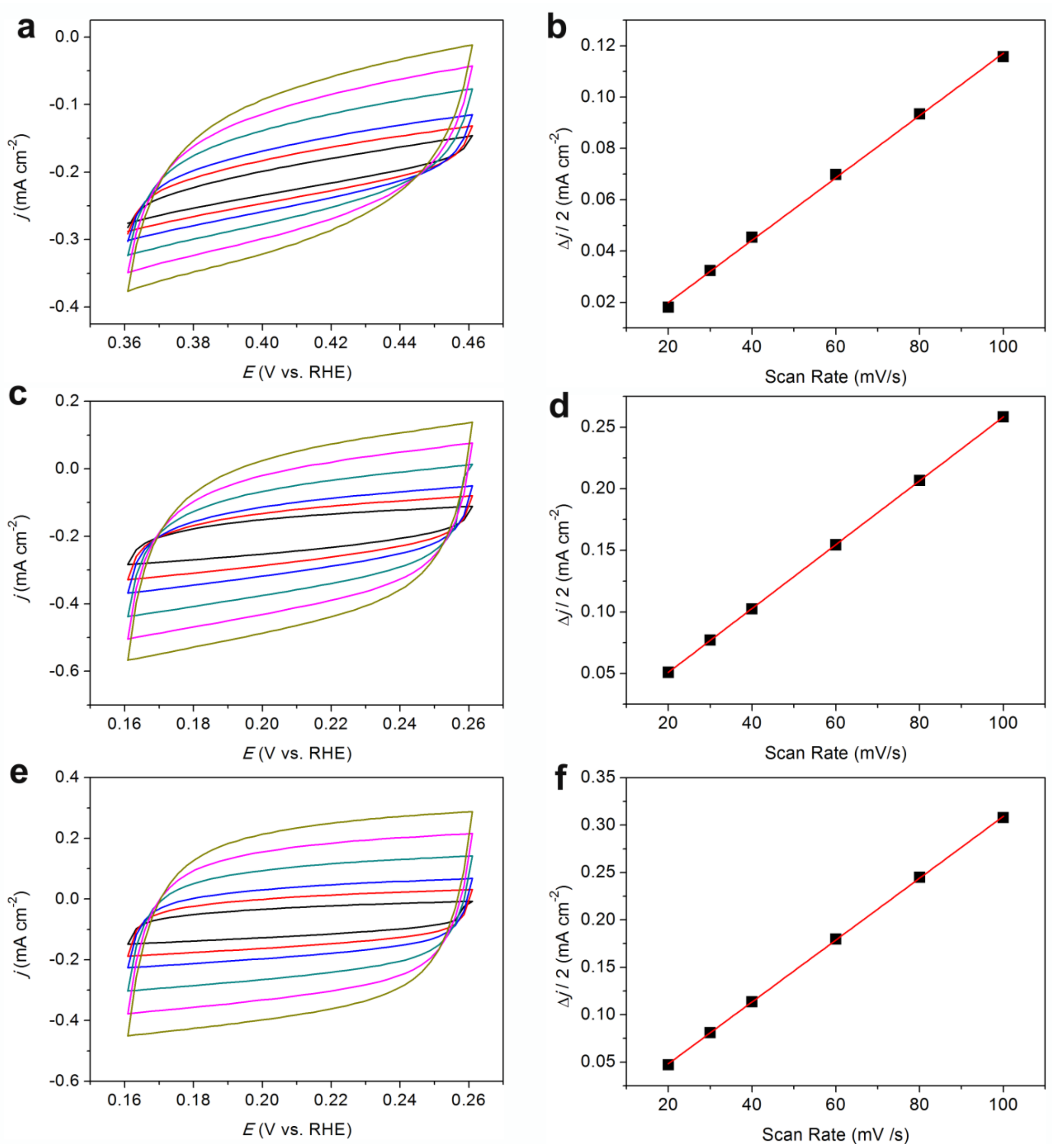

Supplementary Fig. 25. Electrochemical surface area measurement. a-f, Cyclic voltammagrams taken over a range of scan rates and determination of double-layer capacitance on Cu NP (**a, b**), Cu NB (**c, d**) and Cu AD (**e, f**) electrocatalysts in 1M KOH.

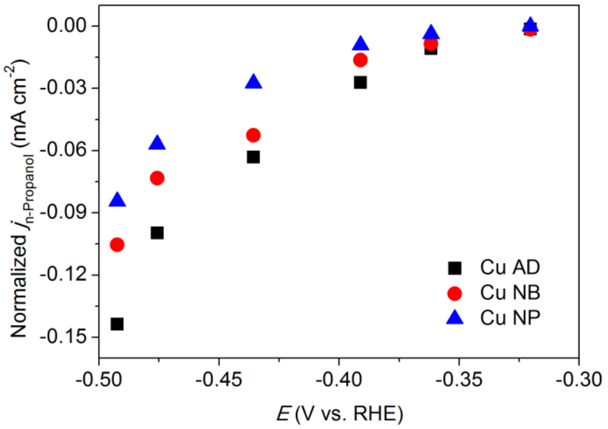

Supplementary Fig. 26. Electrochemical surface area normalized partial currents of n-propanol on different electrocatalysts.

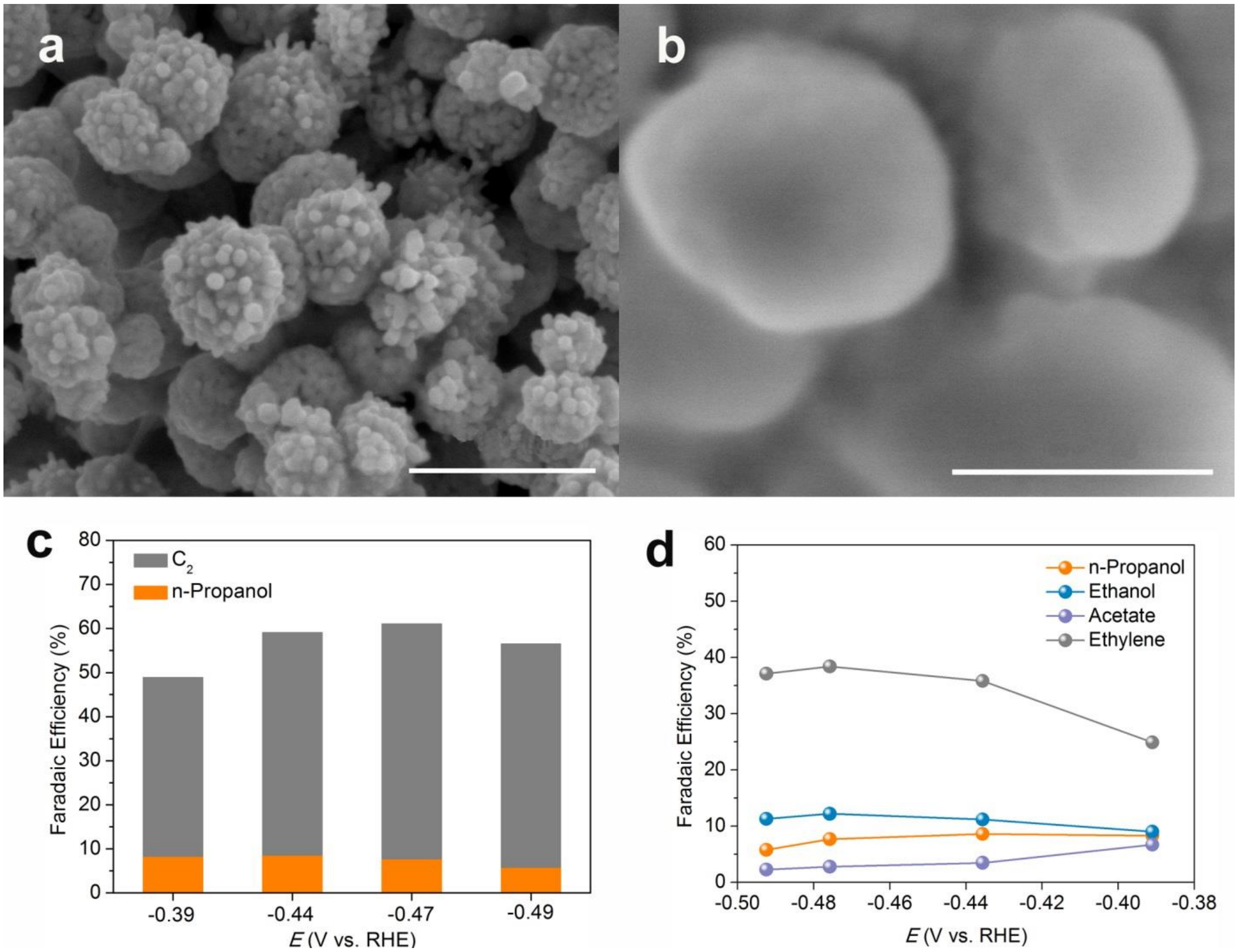

Supplementary Fig. 27. Morphology and CO-RR performance of Cu AD after thermal annealing. a, b, SEM images of Cu AD post-annealed at 150 ℃ for 2h under N_2. The scale bars are 500 nm in (**a**) and 50 nm in (**b**). **c, d,** CO-RR product distributions of Cu AD after thermal annealing.

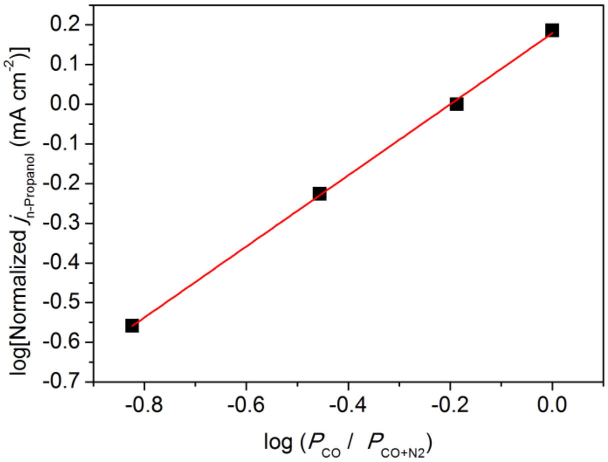

Supplementary Fig. 28. Plot of the partial current density for n-propanol as a function of the CO partial pressure on Cu adparticle electrocatalysts at -0.36 V vs. RHE, a slope value of ~1 is obtained.

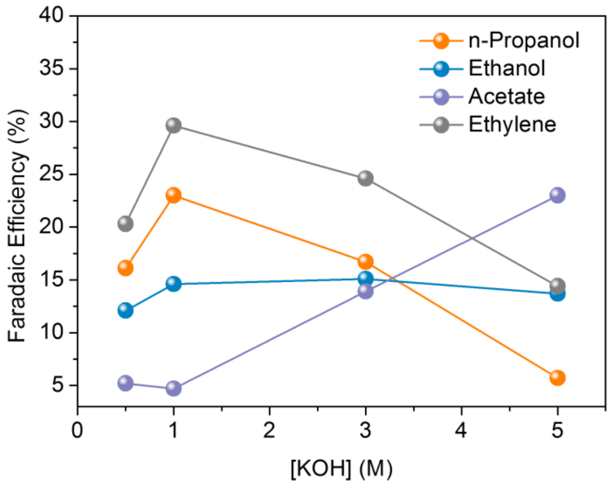

Supplementary Fig. 29. CO-RR product selectivities (FE, %) on Cu adparticle electrocatalysts in different [KOH] electrolytes at ~-0.47 V vs. RHE.

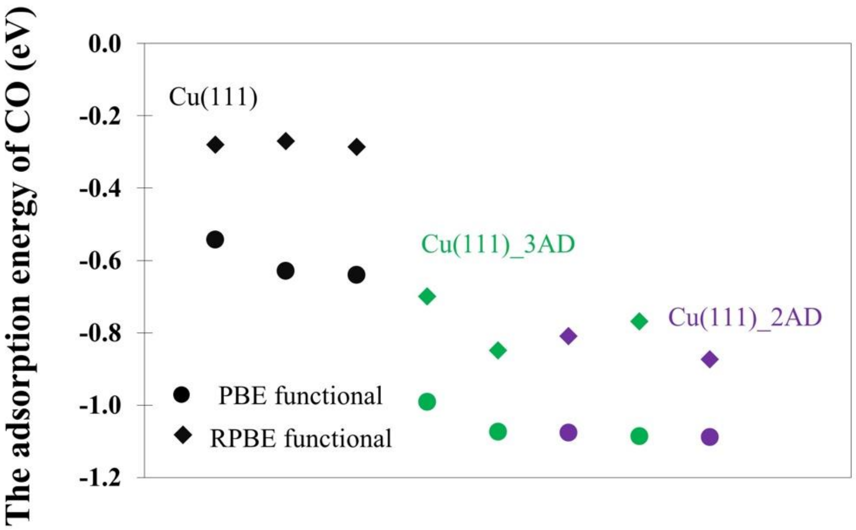

Supplementary Fig. 30. The adsorption energies of CO over Cu(111) in the presence and absence of Cu adparticles using PBE and RPBE functionals.

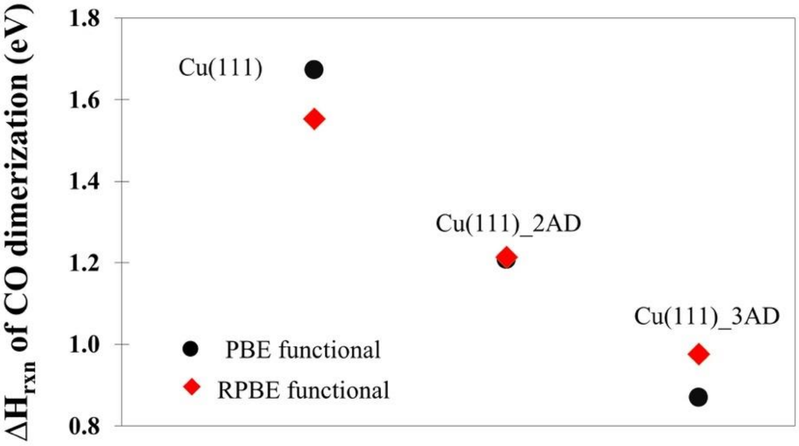

Supplementary Fig. 31. The reaction energies of CO dimerization over Cu(111) in the presence and absence of Cu adparticles using PBE and RPBE functionals.'

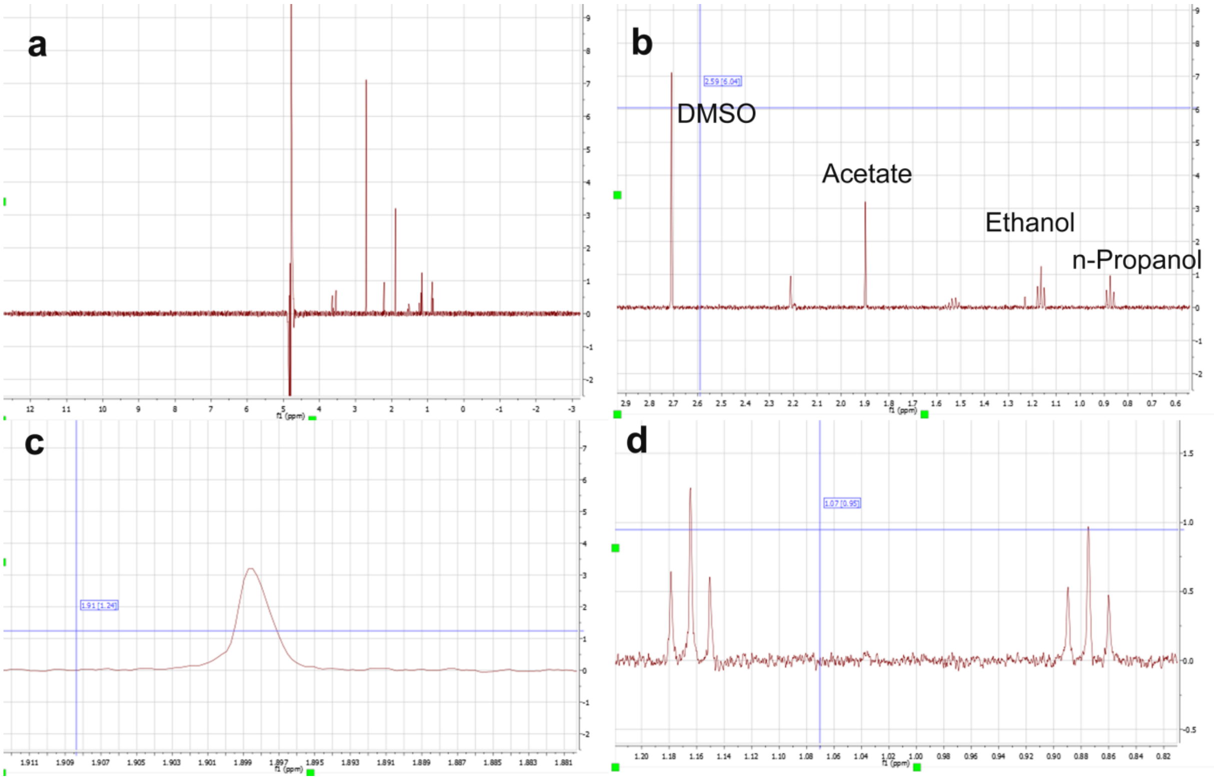

Supplementary Fig. 32. NMR analysis. a-d, Representative ^{1}H-NMR spectrum CO-RR on Cu adparticle electrocatalysts at -0.44V vs. RHE in 1M KOH. DMSO (Dimethyl sulphoxide) was used as an internal for liquid products quantification.

Supplementary Table 1. The coordination number (CN) of surface Cu / Cu adparticles in the examined surfaces.

Surfaces	Position of Cu	CN
Cu(111)	Surface Cu	9
Cu(111)_2AD	Cu_AD	4
Cu(111)_3AD	Cu_AD	5
Cu(100)	Surface Cu	8
Cu(100)_2AD	Cu_AD	5
Cu(100)_4AD	Cu_AD	6
Cu(211)	Cu step atom	7
	Cu upper terrace atom	9
	Cu lower terrace atom	10
Cu(211)_2AD	Cu_AD	3

Supplementary Table 2. The adsorption energies of CO on various Cu surfaces in the presence and absence of the Cu adparticles.

Surfaces	Adsorption sites	E_{ad} (eV)
Cu(111)	top	-0.543
	fcc	-0.640
	hcp	-0.629
Cu(111)_2AD	top	-1.088
	bridge	-1.076
Cu(111)_3AD	top	-1.073
	bridge	-0.991
	hollow	-1.085
Cu(100)	top	-0.813
	bridge	-0.795
Cu(100)_2AD	top	-0.968
	bridge	-0.865
Cu(100)_4AD	top	-0.938
	bridge	-0.858
	bri_near_ad	-0.891
Cu(211)	st_t1_bri	-0.628
	st	-0.937
	sb	-0.963
	t1_t2_hollow	-0.704
	st_t2_hollow	-0.847
Cu(211)_2AD	bri	-0.980
	top	-1.084

Supplementary Table 3. The reaction energies (ΔH_{rxn}) of CO=CO on various Cu surfaces in the presence and absence of Cu adparticles.

Surfaces	2CO co-adsorption site	*OCCO adsorption site	ΔH_{rxn} (eV)
Cu(111)	fcc_hcp	fcc	1.672
Cu(111)_2AD	briad_top	bri	1.208
Cu(111)_3AD	briad_topad	hollow	0.870
Cu(100)	2top	hollow	0.845
Cu(100)_2AD	topnearad_bri	bri	0.962
Cu(100)_4AD	hollowad_topad	hollowad	0.844
Cu(211)	2sb	sb	1.445
Cu(211)_2AD	briad_t1t2hollow	sbad	1.256

Supplementary Table 4. The relative adsorption energies (E_{ad}') of the most favorable configuration of $*CCH_2$ species on Cu(111) in the presence and absence of Cu adparticles. All the adsorption energies of $*CCH_2$ are reference to the one on Cu(111).

Surfaces	$*CCH_2$ adsorption site	E_{ad}' (eV)
Cu(111)	fcc	0.000
Cu(111)_2AD	hollow_near_ad	-0.431
Cu(111)_3AD	briad	-0.782

Supplementary Table 5. The relative adsorption energies ($E_{ad}{}'$) of the most favorable configuration of *OCCOH species on Cu(111) in the presence and absence of Cu adparticles. All the adsorption energies of *OCCOH are referred to the one on Cu(111).

Surfaces	*OCCOH adsorption site	$E_{ad}{}'$ (eV)
Cu(111)	bri	0.000
Cu(111)_2AD	briad	-0.993
Cu(111)_3AD	briad	-0.730

Supplementary Table 6. The reaction energies (ΔH_{rxn}) of CO-OCCOH coupling on various Cu surfaces in the presence and absence of Cu adparticles.

Surfaces	CO-OCCOH co-adsorption site	*OCOCCOH adsorption site	ΔH_{rxn} (eV)
Cu(111)	hcp_fcc	fcc	0.359
Cu(111)_2AD	briad_top	bri	0.457
Cu(111)_3AD	briad_topad	hollow	0.131
Cu(100)	hollow_ad	hollow	0.349
Cu(100)_2AD	briad_bri	bri	0.013
Cu(100)_4AD	hollowad_top	hollowad	-0.099
Cu(211)	hollow_sb	sb	0.125
Cu(211)_2AD	sbad_st	sbad	-0.399

Supplementary Table 7. The reaction energies (ΔH_{rxn}) of CO-CCH_2 coupling on various Cu surfaces in the presence and absence of Cu adparticles.

Surfaces	CO-CCH_2 co-adsorption site	*$OCCCH_2$ adsorption site	ΔH_{rxn} (eV)
Cu(111)	hcp_hcp	fcc	-0.148
Cu(111)_2AD	briad_hcp	bri	-0.770
Cu(111)_3AD	hollowad_hcp	hollowad	-0.588
Cu(100)	hollow_bri	hollow	-0.114
Cu(100)_2AD	2topad	briad	-0.588
Cu(100)_4AD	hollowad_topad	hollowad	-0.239
Cu(211)	hollow_sb	st_t1_hollow	0.045
Cu(211)_2AD	sbad_st	sbad	-0.720

Supplementary Table 8. Faradaic efficiencies of products distribution of three sets of electrocatalysts at different applied potentials in 1 M KOH under a CO gas condition.

Sample	Applied potential (V vs. RHE)	$FE_{Hydrogen}$ (%)	$FE_{Ethylene}$ (%)	$FE_{Acetate}$ (%)	$FE_{Ethanol}$ (%)	$FE_{N\text{-}propanol}$ (%)	FE_{Total} (%)
Cu AD	-0.32	30.1±2.2	4.7±0.3	17.6±0.4	12.2±0.3	2.8±0.2	~67.4
	-0.36	25.6±1.4	15.6±0.8	9.7±0.4	15.3±0.5	14.6±0.6	~80.8
	-0.39	22.1±0.8	24.7±0.6	7.5±0.5	14.3±1.4	21.1±1.7	~89.7
	-0.44	17.6±1.2	29.6±0.5	4.7±0.6	14.6±1.5	23±1.5	~89.5
	-0.47	15±1.1	35.5±2.2	4.8±0.3	17.4±2	23.1±2	~95.8
	-0.49	12.3±0.5	34.3±1.7	5.5±0.1	17.6±0.4	19.9±1.3	~89.6
	-0.51	9.5±0.2	39.1±1.6	5.7±0.2	17.5±0.1	15.6±0.7	~87.4
	-0.56	9.9±0.8	39±0.9	6±0.2	18.7±0.3	14.7±0.2	~88.3
	-0.61	8.6±0.3	38.7±0.5	7.1±0.3	21±1	11.4±0.1	~86.8
	-0.66	4.8±0.2	43.1±2.1	6.5±0.4	28±0.2	11.5±0.4	~93.9
Cu NB	-0.32	29.8±0.6	3.8±0.2	17.8±0.9	12.5±0.6	2.2±0.1	~66.1
	-0.36	23.8±1.2	11.9±0.3	15.5±2.5	11.1±1.5	9.1±1.4	~71.4
	-0.39	18.4±0.2	22.5±2.2	9.2±1.4	11.9±2	11.9±0.8	~73.9
	-0.44	12.5±0.3	30.8±0.9	8.3±0.6	14.6±0.3	16.2±1.6	~82.4
	-0.47	13±0.9	36.3±1.6	6.4±0.8	14±0.6	14.1±1.5	~83.8
	-0.49	11.4±0.2	42.1±1.2	7.2±0.8	13.5±1.1	12.3±1	~86.5
Cu NP	-0.32	74.4±2.3	2.8±0.1	22.6±1.2	0	0	~99.8
	-0.36	37.7±1.5	11.2±0.8	6.9±0.2	5.2±0.3	4.6±0.2	~65.6
	-0.39	34.5±2.1	19.8±0.5	5.5±0.2	11.7±0.6	7.7±0.4	~79.2
	-0.44	23.5±0.3	32±0.4	5.9±0.4	12.2±1.2	11.1±1	~84.7
	-0.47	18.9±1	32.7±0.5	3.9±0.5	12.6±0.9	12.1±0.3	~80.2
	-0.49	16.9±0.5	37.7±0.6	6.2±1	16.7±0.5	7.9±0.5	~85.4
	-0.51	16±0.8	34.9±1.1	4.8±0.7	16.7±1	7.4±0.5	~79.8
	-0.56	14.9±0.2	35.4±1.4	9.8±0.3	19.4±1	4.8±0.3	~84.3

Supplementary Table 9. Measured capacitance values and surface roughness factors of various electrode materials.

Electrode	Capacitance	Surface roughness factor
Polycrystalline Cu	29 μF	1
Cu NPs	1.21 mF	42
Cu NBs	2.59 mF	89
Cu ADs under 100%CO	3.26 mF	112

The surface roughness factor for polycrystalline Cu is defined to be 1 *(1)*.

Supplementary Table 10. Summary of CO_2/CO-RR performances on different Cu-based electrocatalysts for n-propanol generation.

Electrocatalyst	Reaction Type	Electrolyte	$FE_{n\text{-}propanol}$	$j_{n\text{-}propanol}$	Potential vs. RHE (V)	Reference
**Cu AD**	**CO-RR**	**1 M KOH**	**23%**	**11.16**	**-0.47**	**This work**
**Cu NB**	**CO-RR**	**1 M KOH**	**16.2%**	**4.68**	**-0.44**	**This work**
**Cu NP**	**CO-RR**	**1 M KOH**	**12.1%**	**2.39**	**-0.47**	**This work**
1. Oxide-derived Cu	CO-RR	0.1 M KOH	10%	0.0812	-0.4	*(1)*
2. Densely-packed Cu	CO_2-RR	0.1 M $KHCO_3$	5.9%	0.755	-0.95	*(2)*
3. Activated Cu mesh	CO_2-RR	0.5 M $KHCO_3$	13.1%	1.33	-0.9	*(3)*
4. Agglomerated Cu	CO_2-RR	0.1 M $KHCO_3$	8.75%	1.74	-0.95	*(4)*

Supplementary Methods

Computational details. To demonstrate the low-coordination effect on C_3 formation during CO-RR, we examined various under-coordinated Cu sites on the Cu(111) and Cu(100) surfaces with the inclusion of different number of Cu adatoms (**Supplementary Fig. 1**). For Cu(111), we have examined the pure Cu(111) surface, the Cu(111) surface with two Cu adatoms (Cu(111)_2AD), three Cu adatoms (Cu(111)_3AD). For Cu(100), we have examined the pure Cu(100) surface, the Cu(100) surface with two Cu adatoms (Cu(100)_2AD), four Cu adatoms (Cu(100)_4AD). For Cu(211), we have examined the pure Cu(211) surface and the Cu(211) surface with two Cu adatoms (Cu(211)_2AD). In this way, we can have various situations of coordination number of surface Cu atoms (**Supplementary Table 1**).

The adsorption energy of a CO molecule over a Cu surface in the presence and absence of Cu adparticles (**Fig. 1a**) was calculated by Equation 1:

$$E_{ad} = E_{(CO/slab)} - E_{(slab)} - E_{(CO(gas))} \quad (1)$$

where $E_{(CO/slab)}$ represents the total energy for a CO molecule over the Cu slab; $E_{(slab)}$ is the total energy of the bare slab and $E_{(CO(gas))}$ is the carbon monoxide gas phase energy. Here, the more negative value of the adsorption energy represents a stronger binding strength of the CO molecule. The optimized geometries of various CO adsorption over various Cu surfaces (**Fig. 1a**) are presented in **Supplementary Fig. 2-4** and their corresponding adsorption energies are given in **Supplementary Table 2**.

The reaction energy (ΔH_{rxn}) of the CO=CO dimerization was given by Equation 2:

$$\Delta H_{rxn} = E_{(OCCO^*)} - E_{(2CO^*)} \quad (2)$$

where $E_{(OCCO*)}$ represents the total energy for a *OCCO intermediate over the slab; $E_{(2CO^*)}$ is the total energy of two CO molecules co-adsorption over the slab. Here, the more negative value

of the reaction energy represents a more exothermic CO=CO coupling reaction. The optimized geometries of various two *CO co-adsorption and *OCCO adsorption over various Cu surfaces (**Fig. 1b**) are presented in **Supplementary Fig. 5-6** and their corresponding CO=CO coupling reaction energies are given in **Supplementary Table 3**.

To check the stability of *C_2 intermediates (i.e., *CCH_2 and *OCCOH) on various Cu surfaces, the relative adsorption energy ($E_{ad}{}'$) of *CCH_2 or *OCCOH species over a Cu surface in the presence and absence of Cu adparticles was calculated by Equation 3:

$$E'_{ad} = (E_{(C2/Cu(111)\_ad)} - E_{(Cu(111)\_ad)}) - (E_{(C2/Cu(111))} - E_{(Cu(111))}) \quad (3)$$

where ($E_{(C2/Cu(111)\_ad)}$ represents the total energy for a *C_2 intermediate over the Cu(111) surface with Cu adparticles; $E_{(Cu(111)\_ad)}$ is the total energy of the bare slab - the Cu(111) surface with Cu adparticles. $E_{(C2/Cu(111))}$ stands for the total energy for a *C_2 intermediate over a pure Cu(111) surface and $E_{(Cu(111))}$ stands for the total energy for a pure Cu(111) surface. Here, the more negative value of the relative adsorption energy represents a stronger binding strength of the *C_2 intermediate.

The reaction energy (ΔH_{rxn}) of the CO-OCCOH coupling was given by Equation 4:

$$\Delta H_{rxn} = E_{(OCOCCOH^*)} - E_{(CO\_OCCOH^*)} \quad (4)$$

where $E_{(OCOCCOH^*)}$ represents the total energy for a *OCOCCOH intermediate over the slab; $E_{(CO\_OCCOH^*)}$ is the total energy of *CO and *OCCOH species co-adsorption over the slab. Here, the more negative value of the reaction energy represents a more exothermic CO-OCCOH coupling reaction. The optimized geometries of *CO and *OCCOH co-adsorption and *OCOCCOH adsorption over various Cu surfaces (**Fig. 1c**) are presented in **Supplementary Fig. 7-8** and their corresponding CO-OCCOH coupling reaction energies are given in **Supplementary Table 6**.

The reaction energy (ΔH_{rxn}) of the CO-CCH_2 coupling was given by Equation 5:

$$\Delta H_{rxn} = E_{(OCCCH_2^*)} - E_{(CO\_CCH_2{}^*)} \quad (5)$$

where $E_{(OCCCH_2^*)}$ represents the total energy for a *$OCCCH_2$ intermediate over the slab; $E_{(CO\_CCH_2{}^*)}$ is the total energy of *CO and *CCH_2 species co-adsorption over the slab. Here, the more negative value of the reaction energy represents a more exothermic CO-CCH_2 coupling reaction. The optimized geometries of *CO and *CCH_2 co-adsorption and *$OCCCH_2$ adsorption over various Cu surfaces (**Fig. 1d**) are presented in **Supplementary Fig. 9-10** and their corresponding CO-CCH_2 coupling reaction energies are given in **Supplementary Table 7**.

Furthermore, we applied the Neugebauer and Scheffler method to add the applied field and charging surface to examine how the applied field can influence the CO adsorption energies, the reaction energies of CO dimerization and CO-OCCOH coupling over the Cu(111) surface in the presence and absence of Cu adparticles.

As shown in **Supplementary Fig. 11-13**, by increasing the applied field from 0 to 0.8 V/Å, the adsorption energies of CO molecule on a Cu surface at their most favourable adsorption sites monotonically increased, and the reaction energies of CO dimerization / C_1-C_2 couplings over a Cu surface monotonically decreased.

In addition, the RPBE functionals are found to perform well for the adsorption energies of the catalytic intermediates on single-crystal late transition-metal surfaces that involve pure chemisorption (i.e., CO over Cu). In this work, we also compared both PBE functional and RPBE functional on the adsorption of CO as well as the reaction energy of the CO dimerization.

As shown in **Supplementary Fig. 30**, the GGA-PBE functional overestimated the adsorption energies of CO over Cu with various Cu adparticles. But similar trend can be found using both RPBE and PBE functionals, in which the Cu adparticles can significantly increase the CO

adsorption energy. Thus, the main conclusion that Cu adparticles enhance the CO adsorption energy still holds true. As shown in **Supplementary Fig. 31**, the difference in the reaction energies of CO dimerization between PBE functional and RPBE functional is less than 0.1 eV. And the main conclusion that Cu adparticles enhance the CO dimerization holds true using RPBE functional as well.

Supplementary References

1 Li, C. W., Ciston, J. & Kanan, M. W. Electroreduction of carbon monoxide to liquid fuel on oxide-derived nanocrystalline copper. *Nature* **508**, 504-507 (2014).

2 Kim, D., Kley, C. S., Li, Y. F. & Yang, P. D. Copper nanoparticle ensembles for selective electroreduction of CO_2 to C_2-C_3 products. *Proc. Natl. Acad. Sci. U. S. A.* **114**, 10560-10565 (2017).

3 Rahaman, M., Dutta, A., Zanetti, A. & Broekmann, P. Electrochemical reduction of CO_2 into multicarbon alcohols on activated Cu mesh catalysts: An identical location (IL) study. *ACS Catal.* **7**, 7946-7956 (2017).

4 Ren, D., Wong, N. T., Handoko, A. D., Huang, Y. & Yeo, B. S. Mechanistic insights into the enhanced activity and stability of agglomerated Cu nanocrystals for the electrochemical reduction of carbon dioxide to n-propanol. *J. Phys. Chem. Lett.* **7**, 20-24 (2016).
